# Effects of shading on morphology, photosynthetic characteristics, and yield of different *Amorphophallus* varieties

**DOI:** 10.3389/fpls.2026.1931411

**Published:** 2026-08-24

**Authors:** Jinwei Li, Wenxian Xu, Minghui Chen, Yanxiong Gong, Yanwei Ruan, Zesen Lai, Ruyue Xu, Huifang Yuan, Xiangshuai Yan

**Affiliations:** Yunnan Institute of Tropical Crops, Jinghong, China

**Keywords:** chlorophyll fluorescence, comprehensive evaluation, inter-varietal differences, konjac, photosynthetic characteristics, shade tolerance, shading, yield

## Abstract

Konjac (*Amorphophallus* spp.) is a typical shade-demanding cash crop, yet the adaptability of different varieties to light intensity remains poorly understood. This study used one *Amorphophallus konjac* variety (HMY01) and seven *Amorphophallus bulbifer* varieties (YunRe1701, YunRe1707, YunRe1709, YunRe1710, YunRe1726, XD09, and GY02) as materials, and established five light gradients (100%, 70%, 50%, 25%, and 7% of natural light) to systematically measure morphological, photosynthetic, and yield-related traits. The results showed that: (1) Moderate shading (25%–50% light) significantly promoted morphological growth, photosynthetic efficiency, and corm yield in most varieties, while both full light and 7% extreme low light inhibited growth and development, with significant differences in optimal light intensity among varieties. (2) Cluster analysis classified the eight varieties into three functional groups: HMY01 (high photosystem II (PSII) stability), morphology-dominant type (YunRe1701, XD09, GY02, emphasizing plant height and leaf area expansion), and photosynthesis-dominant type (YunRe1707, YunRe1709, YunRe1710, YunRe1726, emphasizing light energy use efficiency). (3) Principal component analysis (PCA) extracted four principal components with a cumulative contribution rate of 81.23%, effectively distinguishing two synergistic dimensions: ‘light energy utilization and carbon assimilation’ and ‘morphological construction and pigment adjustment’. This study provides a scientific basis for variety selection and light management in konjac understory intercropping systems.

## 1 Introduction

Light is the energy source that drives plant photosynthesis and a key environmental factor regulating plant morphogenesis, carbon assimilate partitioning, and yield formation (Wang et al., 2024). In natural habitats, light intensity varies dynamically with canopy closure, cloud movement, and seasonal changes, and plants have developed physiological and ecological characteristics adapted to their light environments over long-term evolution (Zhang et al., 2021). Based on their light requirements, plants can be classified into two major groups: sun plants and shade plants (Augspurger, 1984). Shading directly affects leaf light energy capture efficiency, carbon assimilation capacity, and the partitioning of assimilates between sources (leaves) and sinks (harvest organs) by reducing photosynthetically active radiation, thereby influencing crop growth, development, and yield formation (Wang et al., 2024; Valladares and Niinemets, 2008; Liu et al., 2026). Under cultivated conditions, light management has become one of the key agronomic measures affecting crop yield and quality. For shade-demanding crops that originated from understory environments, determining their optimal light intensity and the differences among varieties has significant theoretical and practical implications.

Konjac (*Amorphophallus* spp.) is a perennial herbaceous plant of the genus *Amorphophallus* in the family Araceae. Its underground corms are rich in konjac glucomannan, making it a specialty cash crop of considerable economic value (Liu, 2004). Plants of the genus *Amorphophallus* originated from understory environments in tropical and subtropical regions and have developed shade-loving and shade-tolerant physiological and ecological characteristics over long-term evolution (Yuan et al., 2025a). *Amorphophallus bulbifer* is characterized by high yield and strong resistance, and its cultivation area in China ranks third, following *Amorphophallus konjac* and *Amorphophallus albus.* However, different konjac species or varieties exhibit significant differences in their tolerance to light intensity. Studies have shown that although *A. konjac* is intolerant to strong light, it can be grown in monoculture in agricultural production and is considered to have a certain capacity for high-light tolerance. In contrast, *Amorphophallus xiei* is rarely grown in monoculture and is mostly intercropped under forest canopies or with other tall-stalk crops, making it a typical shade-demanding plant (Han et al., 2013; Ma et al., 2026). Han et al. (2013) compared the photosynthetic characteristics and photoprotective mechanisms of *A. konjac* and *A. xiei* under three light gradients (100%, 15%, and 2% light transmittance) and found that *A. konjac* exhibited a higher maximum photosynthetic rate (Amax) under full light, while *A. xiei* reached its maximum Amax under 15% light transmittance, suggesting that the high-light tolerance of *A. konjac* may stem from its relatively high photosynthetic capacity and effective photoprotective mechanisms. Ma et al. (2026) confirmed in rubber plantation intercropping systems of different tree ages that *A. xiei* exhibited stronger photosynthesis and faster photosynthetic induction rates under medium light (30%–45% transmittance) and low light (10%–15% transmittance), and could protect photosystem II (PSII) from photoinhibition by increasing non-photochemical quenching (NPQ), whereas photoinhibition under high light stress (65%–75% transmittance) was the primary cause of its inability to grow normally. Qin et al. (2019) used *A. konjac* cv. No.1 Chumohua as material and set shading rates of 0%, 50%, 70%, and 90%, finding that photosynthetic capacity and corm yield were highest under 50%–70% shading, further confirming the promoting effect of moderate shading on konjac growth. These studies indicate that plants of the genus *Amorphophallus* possess typical shade-loving characteristics, but significant differentiation exists among different species/varieties in shade tolerance, photosynthetic capacity, and photoprotective strategies.

The effects of shading on plant morphology, photosynthetic physiology, and yield have been extensively studied in various crops. At the morphological level, plants typically enhance light interception capacity under shading conditions by increasing plant height and expanding leaf area (Ren et al., 2024; Zhang et al., 2023; Wu et al., 2023). Wang et al. (2024) conducted a comparative study on shade-tolerant and shade-sensitive peanut varieties and found that shading caused a significantly smaller increase in main stem height in shade-tolerant varieties compared with shade-sensitive ones, while the reductions in leaf area and dry matter accumulation were also more moderate, indicating that morphological plasticity is an important indicator of inter-varietal differences in shade tolerance. At the photosynthetic physiological level, plant responses to shading involve multi-dimensional adjustments in chlorophyll content and composition, light response curve characteristics, and chlorophyll fluorescence parameters. In general, plants enhance their weak-light capture capacity under shading by increasing the relative content of chlorophyll b (reducing the Chl a/b ratio), but excessive shading reduces net photosynthetic rate (Pn), stomatal conductance (Gs), and transpiration rate (Tr) (Eskandarzade et al., 2023). Chlorophyll fluorescence parameters such as the maximum photochemical efficiency of PSII (Fv/Fm), actual photochemical efficiency (Y(II)), NPQ, photochemical quenching (qL), and electron transport rate (ETR) are highly sensitive to changes in light intensity and serve as effective probes for rapidly assessing photosynthetic apparatus function and photoinhibition severity (Qin et al., 2019; Shi et al., 2025; Yang et al., 2019; Maxwell and Johnson, 2000). At the yield level, shading determines economic yield by influencing the photosynthetic assimilatory capacity of source organs and the carbohydrate supply to sink organs. Liu et al. (2026), in a shading experiment during the ear stage of summer maize, found that shading led to a 65.00% decrease in leaf sucrose content, a 39.50% reduction in plant biomass, and significant decreases in soluble sugars and starch contents in both tassels and ears, ultimately resulting in a 32.20% reduction in kernel number per ear and a 32.80% decrease in yield per plant, revealing the causal chain of ‘photosynthetic decline—insufficient source supply—inhibited sink development—yield reduction’. Di et al. (2025) conducted a comprehensive evaluation of shade tolerance in 15 water lily varieties and found significant differentiation in shade tolerance among varieties, with some varieties still flowering normally under 45%–74% shading while sensitive varieties completely failed to flower under severe shading, further confirming that the differentiation of shade-tolerance strategies among varieties is a universal feature of plant adaptation to light environments.

Although extensive research has been conducted on the effects of shading on crop photosynthetic physiology and yield, several scientific questions remain to be addressed. First, existing studies on konjac shading have mostly focused on single or a few varieties of *A. konjac* and *A. xiei*, lacking comparative studies on multiple konjac varieties (especially *A. bulbifer* varieties with different genetic backgrounds) under systematic light gradients. Second, previous studies have tended to focus separately on morphology, photosynthesis, or yield, with insufficient systematic analysis of the coupling relationships among ‘morphology-photosynthesis-yield’, and in particular, a lack of research on inter-varietal shade-tolerance strategy typing based on multi-indicator comprehensive evaluation (e.g., principal component analysis and cluster analysis). Third, the physiological mechanisms underlying the differences in shade tolerance among konjac varieties remain unclear.

To address these research gaps, this study used eight konjac varieties (including one *A. konjac* variety, HMY01, and seven *A. bulbifer* varieties: YunRe1701, YunRe1707, YunRe1709, YunRe1710, YunRe1726, XD09, and GY02) as materials and established five light gradients (light transmittance of 100%, 70%, 50%, 25%, and 7%) to systematically investigate the effects of shading on morphological traits, photosynthetic physiological indices, chlorophyll fluorescence parameters, and yield of different konjac varieties. The following hypotheses were proposed: (1) Moderate shading (25%–50% of natural light transmittance) can significantly promote photosynthetic efficiency and yield in most konjac varieties, but the optimal light intensity varies among varieties; (2) The eight konjac varieties can be classified into different functional groups based on their shade-tolerance strategies, with significant interspecific differentiation between *A. konjac* (HMY01) and *A. bulbifer* varieties, and within *A. bulbifer*, further differentiation into types that emphasize morphological adjustment or photosynthetic optimization; (3) Yield is significantly correlated with morphological and photosynthetic indices, and the combined use of principal component analysis and membership function analysis on multiple indicators can effectively evaluate the shade-tolerance capacity and suitable light environment of different varieties. The results of this study will reveal the differentiation patterns of shade-tolerance strategies among konjac varieties and provide a scientific basis for variety selection and light management in understory intercropping systems of konjac.

## 2 Materials and methods

### 2.1 Experimental materials and design

The experimental materials used in this study consisted of one *A. konjac* variety (HMY01) and seven *A. bulbifer* varieties (YunRe1701, YunRe1707, YunRe1709, YunRe1710, YunRe1726, XD09, and GY02). All materials were obtained from the Yunnan Institute of Tropical Crops and had been identified in previous studies (Li et al., 2025). The experiment was conducted at the experimental base of the Yunnan Institute of Tropical Crops (22°00′24″N, 100°46′14″E), an altitude of 580 m, an annual average temperature of 23.5°C, an annual rainfall of 1214.9 mm, and an annual sunshine duration of 2126.6 h. It belongs to a tropical monsoon climate with distinct dry and wet seasons. A shading shed was constructed on flat, open terrain with no surrounding obstructions.

The experiment was conducted from May to December 2023 using a split-plot design based on a randomized complete block design (RCBD), with five shading levels as the main plots. Shading nets with nominal shading rates of approximately 30% and 50% were used in single or multiple layers to achieve shading levels of 0%, 30%, 50%, 75%, and 93% (corresponding to light intensities of 100%, 70%, 50%, 25%, and 7% of natural light, respectively). Photosynthetically active radiation (PAR) under different shading treatments was measured on clear days at 3-h intervals using a PAR meter (Spectrum 3415FQF, USA) (**Table 1**). Eight konjac varieties were assigned to the subplots and randomized within each main plot, resulting in a total of 40 treatment combinations, each with 3 block replications. Corms of uniform size and plump shape were selected and planted individually in round plastic pots (one corm per pot) filled with a 1:1 (v/v) mixture of laterite and peat moss. For each treatment combination, 3–4 pots were used as sampling units within each replication, giving a total of 400 pots (5 shading levels × 8 varieties × 3 replications × 3–4 pots per replication). Throughout the experiment, all plants were watered twice a week, each time until the soil was fully saturated. On June 28, each plant received 15 g of compound fertilizer (N:P_2_O_5_:K_2_O = 15:15:15), and on September 20, each plant received an additional 15 g of compound fertilizer (N:P_2_O_5_:K_2_O = 15:5:25) as a top dressing. Weeds were controlled manually during the experimental period.

**Table 1:** Photosynthetically active radiation (PAR) under different shading treatments in different times.

O’clock	Photosynthetically active radiation value (μmol·m^-2^.s^-1^)				
	No shading treatment	30% shading treatment	50% shading treatment	75% shading treatment	93% shading treatment
9:00	1633 ± 17.03	1095 ± 40.78 (67.07%)	836 ± 13.26 (51.22%)	424 ± 17.65 (25.94%)	128 ± 12.35 (7.82%)
12:00	1938 ± 18.00	1276 ± 14.16 (65.82%)	1004 ± 14.41 (51.82%)	472 ± 16.43 (24.35%)	134 ± 16.53 (6.91%)
15:00	1554 ± 139.24	1055 ± 23.93 (67.86%)	777 ± 69.62 (50.00%)	394 ± 31.10 (25.34%)	127 ± 16.14 (8.18%)
18:00	1045 ± 37.36	711 ± 30.90 (68.05%)	514 ± 22.12 (48.17%)	253 ± 12.12 (24.20%)	73 ± 6.17 (6.95)

Values in parentheses indicate the percentage of photosynthetically active radiation (PAR) under each shading treatment relative to that under the unshaded (100% light) treatment, i.e., the relative light intensity. Data are presented as means ± SE (n = 3).

### 2.2 Determination of growth

From August 15 to September 14, 2023 (when aboveground plant morphology had stabilized), healthy plants were selected for measurement of plant height, leaf diameter, and petiole girth. Three biological replicates were performed for each treatment. Plant height was measured as the distance from the base of the petiole to the petiole bifurcation using a tape measure. Leaf diameter was calculated as the average of leaf length and leaf diameter measured with a tape measure. Petiole girth was measured as the circumference at the base of the petiole near the ground surface using a tape measure.

### 2.3 Determination of chlorophyll content

Leaf samples were cut into small pieces, avoiding the main veins. A 0.1 g sample was placed in a 10 mL test tube with 5 mL of 95% ethanol and extracted in the dark until the leaf tissue was completely bleached. The absorbance of the extract was measured at 665 nm and 649 nm using a spectrophotometer. Total chlorophyll content and the chlorophyll a/b ratio were calculated according to established methods (Wang et al., 2024; Zhu et al., 2025). Three biological replicates were performed for each treatment.

### 2.4 Determination of leaf gas exchange parameters

On clear days between 9:00 and 11:00 a.m., leaves of the same orientation were selected, and net photosynthetic rate (Pn), intercellular CO_2_ concentration (Ci), stomatal conductance (Gs), and transpiration rate (Tr) were measured using a portable photosynthesis system (LI-6400, LI-COR, Lincoln, NE, USA) equipped with a red-blue light source leaf chamber. Measurements were conducted at a leaf temperature of 28 °C and a relative humidity of 80%. The CO_2_ concentration in the leaf chamber was set to 400 μmol·mol^-1^, and the PAR intensity was set to 1000 μmol·m^-2^·s^-1^. Measurements were recorded after the readings had stabilized. Three biological replicates were performed for each treatment.

### 2.5 Determination of leaf chlorophyll fluorescence parameters

On clear days between 9:00 and 11:00 a.m., leaves of the same orientation were selected, leaves were dark-adapted for 30 min using dark-adaptation leaf clips (DLC-8), and then chlorophyll fluorescence parameters were measured using a portable modulated chlorophyll fluorometer (PAM-2500, WALZ, Germany). The parameters measured included the maximum photochemical efficiency of photosystem II (Fv/Fm), actual photochemical efficiency (Y(II)), photochemical quenching (qL), non-photochemical quenching (NPQ), and relative electron transport rate (ETR). The actinic light intensity was set to 196 μmol·m^-2^·s^-1^. Three biological replicates were performed for each treatment.

### 2.6 Determination of corm yield

At the konjac maturity stage, after the plants had yellowed and degraded (December 7, 2023), at least three corms per treatment were collected. After removing surface soil, the corms were weighed using an electronic balance (precision: 0.01 g) to calculate the single corm weight per variety.

### 2.7 Statistical analysis

All data are presented as means ± standard errors. One-way ANOVA based on the compare-means model, two-way ANOVA based on the general linear model (GLM), and principal component analysis (PCA) were performed using SPSS 27.0 software (IBM Corp., USA), with mean comparisons conducted using Waller-Duncan test at a significance level of *P* = 0.05. Correlation analysis, cluster analysis, and figure preparation were performed using Origin 2021 software (OriginLab Corp., USA).

#### 2.7.1 Two-way ANOVA

Two-way ANOVA was performed according to the following linear mixed model:


$${\text{Y}}_{\text{ijk}}=\mu +{\text{Bi}}_{+}{\text{Sj}}_{+}{\left(\text{B}\times \text{S}\right)}_{\text{ij}}+{\text{Vk}}_{+}{\left(\text{S}\times \text{V}\right)}_{\text{jk}}+\epsilon_{\text{ijk}}$$

where:

μ = overall mean.

B_i_ = effect of the i-th block (i = 1, 2, 3), random effect.

S_j_ = = effect of the j-th shading treatment (j = 1,…, 5), fixed effect (main-plot factor).

(B×S)_ij_ = block × shading interaction, random effect, used as the error term for testing the main effect of shading (main-plot error).

V_k_ = effect of the k -th variety (k = 1,…, 8), fixed effect (subplot factor).

(S×V)_jk_ = shading × variety interaction, fixed effect.

ϵ_ijk_ = subplot error (residual), random effect, used as the error term for testing the main effect of variety and the shading × variety interaction.

The two-way ANOVA results for the 15 measured traits and the composite index (CPI) are presented in **Supplementary Table 1**.

#### 2.7.2 Principal component analysis

Due to the absence of yield data for HMY01 caused by severe soft rot disease during the later growth stage, principal component analysis (PCA) was performed on the 14 indices excluding yield-related traits across the eight konjac varieties. The number of principal components (PCs) was determined based on the criterion of eigenvalues greater than 1, and four principal components were extracted along with their contribution rates. The comprehensive index values of each principal component were calculated based on the standardized values of the 14 indices and the component loading matrix, using the following formulas:


$$\text{Y}1=-0.14\times {\text{X}}_{\text{Plant height}}-\;0.24\;\times {\text{ X}}_{\text{Leaf diameter}}-\;0.22\;\times {\text{ X}}_{\text{Petiole girth}}-\;0.19\;\times {\text{ X}}_{\text{Total chlorophylly}}+\;0.25\;\times {\text{ X}}_{\text{Chlorophylly a/b}}+\;0.31\;\times {\text{ X}}_{\text{Pn}}+\;0.29\;\times {\text{ X}}_{\text{Gs}}-\;0.17\;\times {\text{ X}}_{\text{Ci}}+\;0.29\;\times {\text{ X}}_{\text{Tr}}+\;0.10\times {\text{ X}}_{\text{Fv/Fm}}+\;0.37\;\times {\text{ X}}_{\text{Y}(\text{II})}-\;0.33\;\times {\text{ X}}_{\text{NPQ}}+\;0.30\;\times {\text{ X}}_{\text{qL}}+\;0.37\;\times {\text{ X}}_{\text{ETR}}$$

$$\text{Y}2\;=\;0.35\;\times {\text{ X}}_{\text{Plant height}}+\;0.43\;\times {\text{ X}}_{\text{Leaf diameter}}+0.27\times {\text{ X}}_{\text{Petiole girth}}+\;0.42\;\times {\text{ X}}_{\text{Total chlorophylly}}-\;0.36\;\times \;X_{\text{Chlorophylly a/b}}+\;0.28\;\times {\text{ X}}_{\text{Pn}}+\;0.22\;\times {\text{ X}}_{\text{Gs}}-\;0.27\;\times {\text{ X}}_{\text{Ci}}+\;0.22\;\times {\text{ X}}_{\text{Tr}}+\;0.19\;\times {\text{ X}}_{\text{Fv/Fm}}+\;0.13\;\times {\text{ X}}_{\text{Y}(\text{II})}\;-\;0.08\;\times {\text{ X}}_{\text{NPQ}}-\;0.01\;\times {\text{ X}}_{\text{qL}}+\;0.09\;\times {\text{ X}}_{\text{ETR}}$$

$$\text{Y}3=-\;0.37\;\times {\text{ X}}_{\text{Plant height}}-\;0.16\;\times {\text{ X}}_{\text{Leaf diameter}}-\;0.33\;\times {\text{ X}}_{\text{Petiole girth}}+\;0.19\times {\text{ X}}_{\text{Total chlorophylly}}-\;0.19\;\times {\text{ X}}_{\text{Chlorophylly a/b}}+\;0.17\;\times {\text{ X}}_{\text{Pn}}+\;0.27\;\times {\text{ X}}_{\text{Gs}}+\;0.21\;\times {\text{ X}}_{\text{Ci}}+\;0.26\;\times {\text{ X}}_{\text{Tr}}+\;0.41\;\times {\text{ X}}_{\text{Fv/Fm}}-\;0.09\;\times {\text{ X}}_{\text{Y(II)}}+\;0.26\;\times {\text{ X}}_{\text{NPQ}}-\;0.42\;\times {\text{ X}}_{\text{qL}}-\;0.14\;\times {\text{ X}}_{\text{ETR}}$$

$$\text{Y}4\;=\;0.03\;\times {\text{ X}}_{\text{Plant height}}+\;0.12\;\times {\text{ X}}_{Leaf\;diameter}+\;0.31\;\times {\text{ X}}_{\text{Petiole girth}}-\;0.28\;\times {\text{ X}}_{\text{Total chlorophylly}}+\;0.29\;\times {\text{ X}}_{\text{Chlorophylly a/b}}+\;0.30\;\times {\text{ X}}_{\text{Pn}}+\;0.40\;\times {\text{ X}}_{\text{Gs}}+\;0.13\;\times {\text{ X}}_{\text{Ci}}+\;0.34\;\times {\text{ X}}_{\text{Tr}}-\;0.37\;\times {\text{ X}}_{\text{Fv/Fm}}-\;0.32\;\times {\text{ X}}_{\text{Y}(\text{II})}+\;0.02\;\times {\text{ X}}_{\text{NPQ}}-\;0.10\;\times {\text{ X}}_{\text{qL}}-\;0.30\;\times {\text{ X}}_{\text{ETR}}$$

where Y_1_, Y_2_, Y_3_, and Y_4_ represent the comprehensive index values of the corresponding principal components, and X represents the standardized value of the corresponding index.

#### 2.7.3 Membership function analysis

(1) The membership function values of the eight konjac varieties were calculated using the following formula:


$$\text{W}\left(\text{Yi}\right)=\frac{\text{Yi}-\text{Ymin}}{\text{Ymax}-\text{Ymin}}$$

where W(Yi) is the membership function value of the i-th comprehensive index; Yi is the value of the i-th comprehensive index; Ymin is the minimum value of the i-th comprehensive index among all varieties; and Ymax is the maximum value of the i-th comprehensive index among all varieties.

(2) The weight of each principal component was calculated using the following formula:


$$\text{P}(\text{Yi})=\frac{\text{Ei}}{\sum_{\text{i}=1}^{\text{n}}\text{Ei}},(\text{i}=1,2,3\ldots \ldots \text{n})$$

where P(Yi) is the weight of the i-th comprehensive index, and Ei is the eigenvalue of the i-th comprehensive index.

(3) The CPI of the eight konjac varieties was calculated using the following formula:


$$\text{CPI}=\sum_{i=1}^{n}\left[\text{W}(\text{Yi})\times \text{P}(\text{Yi})\right],(i=1,2,3\ldots \ldots 4)$$

## 3 Results

### 3.1 Effects of shading on plant morphology of konjac

**Figure 1:**
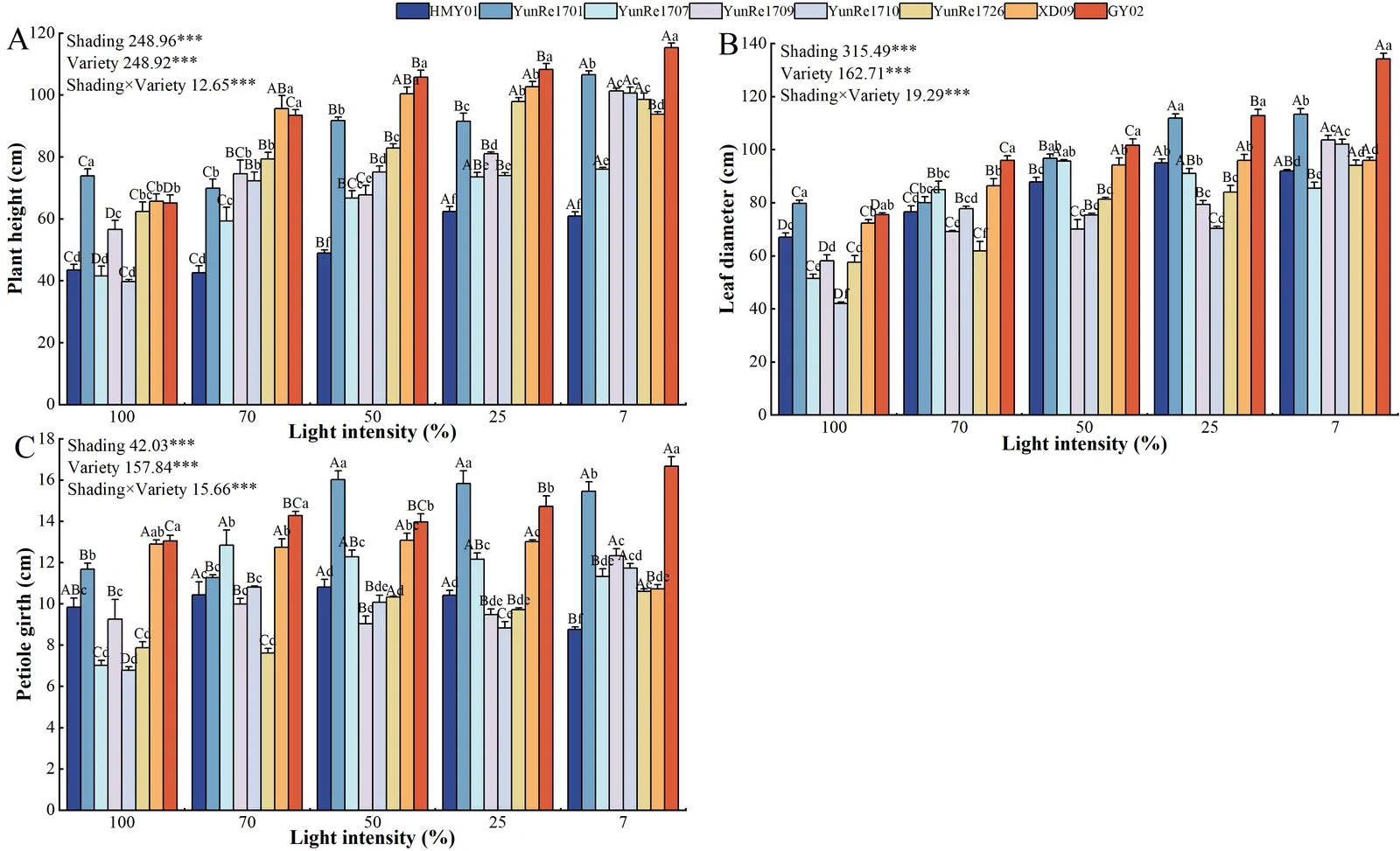
Effects of shading on morphological traits of different konjac varieties. **(A)** plant height, **(B)** leaf diameter, **(C)** petiole girth. Different uppercase letters indicate significant differences among different light treatments for the same variety (*P* < 0.05); different lowercase letters indicate significant differences among different varieties under the same light treatment (*P* < 0.05). *** indicates significance at the *P* < 0.001 level, and the numbers represent the *F*-values. Data are presented as means ± SE (n = 3).

To investigate the effects of shading on aboveground morphology of konjac, we measured plant height, leaf diameter, and petiole girth of different varieties after plant growth had stabilized. Two-way ANOVA revealed that shading, variety, and their interaction all had highly significant effects (*P* < 0.001) on plant height, leaf diameter, and petiole girth (**Figure 1**). Overall, plant height gradually increased with decreasing light intensity (**Figure 1A**). For HMY01, plant height under 50% light was significantly higher than under 100% light (*P* < 0.05, same below), reaching its maximum under 25% light and then stabilizing, with increases of 12.51% and 43.43% compared to 100% light, respectively. For YunRe1701, plant height significantly increased under 50% light and reached its maximum under 7% light, with increases of 24.22% and 44.45% compared to 100% full light, respectively. YunRe1707, YunRe1709, YunRe1710, YunRe1726, and GY02 all showed significant increases in plant height under 25% light, with the highest values observed under 7% light. For XD09, plant height significantly increased under 70% light, reached its maximum under 25% light, and then decreased slightly, with increases of 45.40% and 56.16% compared to 100% full light, respectively.

Leaf diameter also gradually increased with decreasing light intensity (**Figure 1B**). For HMY01, leaf diameter significantly increased under 70% light, reached its maximum under 25% light, and then stabilized, with increases of 14.24% and 41.90% compared to full light, respectively. YunRe1709 and YunRe1726 both showed significant increases under 50% light and reached their maxima under 7% light, with increases of 21.37% and 42.08% for YunRe1709, and 41.35% and 63.56% for YunRe1726 compared to full light, respectively. For YunRe1707, leaf diameter significantly increased under 70% light, reached its maximum under 50% light, and then gradually decreased with further light reduction. YunRe1709, YunRe1710, XD09, and GY02 all showed significant increases in leaf diameter under 70% light and reached their maxima under 7% light.

The effects of shading on petiole girth are shown in **Figure 1C**. For HMY01, YunRe1701, and XD09, petiole girth first increased and then decreased with decreasing light intensity, all reaching their maxima under 50% light. For YunRe1707, petiole girth reached its maximum under 70% light and then gradually decreased, with an increase of 83.02% compared to full light. For YunRe1709, YunRe1710, YunRe1726, and GY02, petiole girth was highest under 7% light, with increases of 33.30%, 73.12%, 34.69%, and 27.84% compared to full light, respectively.

### 3.2 Effects of shading on chloroplast pigment content of konjac

**Figure 2:**
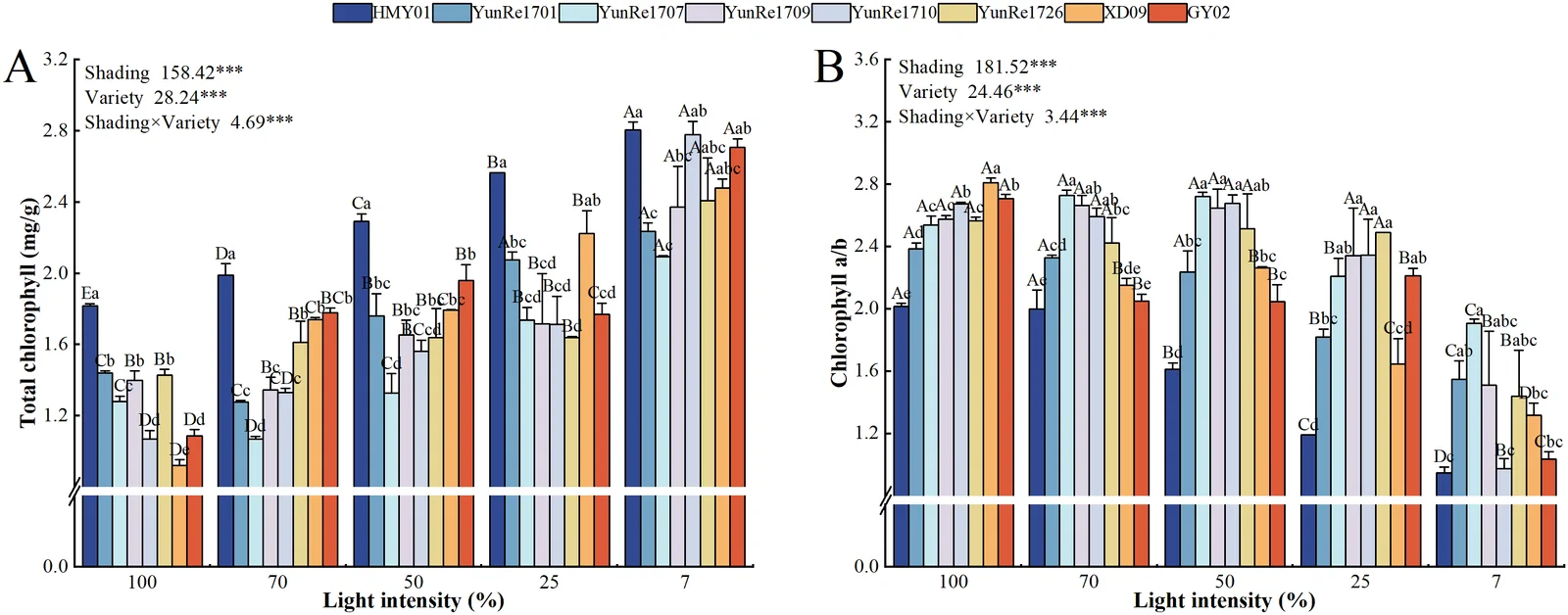
Effects of shading on chlorophyll content of different konjac varieties. **(A)** total chlorophyll, **(B)** chlorophyll a/b. Different uppercase letters indicate significant differences among different light treatments for the same variety (*P* < 0.05); different lowercase letters indicate significant differences among different varieties under the same light treatment (*P* < 0.05). *** indicates significance at the *P* < 0.001 level, and the numbers represent the *F*-values. Data are presented as means ± SE (n = 3).

Two-way ANOVA revealed that shading, variety, and their interaction all had highly significant effects (*P* < 0.001) on leaf chlorophyll content (**Figure 2**). With decreasing light intensity, total chlorophyll content generally exhibited an upward trend across all varieties, while the chlorophyll a/b ratio showed a downward trend. Compared with full light treatment, total chlorophyll content of HMY01 significantly increased by 9.34% under 70% light intensity and became significantly higher than that of other treatments under 7% light intensity; the chlorophyll a/b ratio significantly decreased by 20.30% under 50% light intensity and reached its lowest value, significantly lower than other treatments, under 7% light. For YunRe1701, total chlorophyll content was significantly higher under 50% light intensity than under full light and reached its maximum under 7% light; the chlorophyll a/b ratio significantly decreased under 25% light intensity and reached its minimum under 7% light. For YunRe1707, total chlorophyll content increased by 35.68% under 25% light compared with full light, while the chlorophyll a/b ratio decreased by 12.99% under the same treatment. For YunRe1709, YunRe1710, and YunRe1726, total chlorophyll content was significantly higher under 7% light than under other treatments, and the chlorophyll a/b ratio was significantly lower under 7% light than under other treatments. Compared with full-light treatment, total chlorophyll content of XD09 and GY02 significantly increased under 70% light intensity by 89.13% and 63.80%, respectively; the chlorophyll a/b ratio significantly decreased under 25% light intensity, with reductions of 23.37% and 24.35%, respectively, and reached its minimum under 7% light intensity.

### 3.3 Effects of shading on photosynthesis of konjac

#### 3.3.1 Photosynthetic physiological parameters

**Figure 3:**
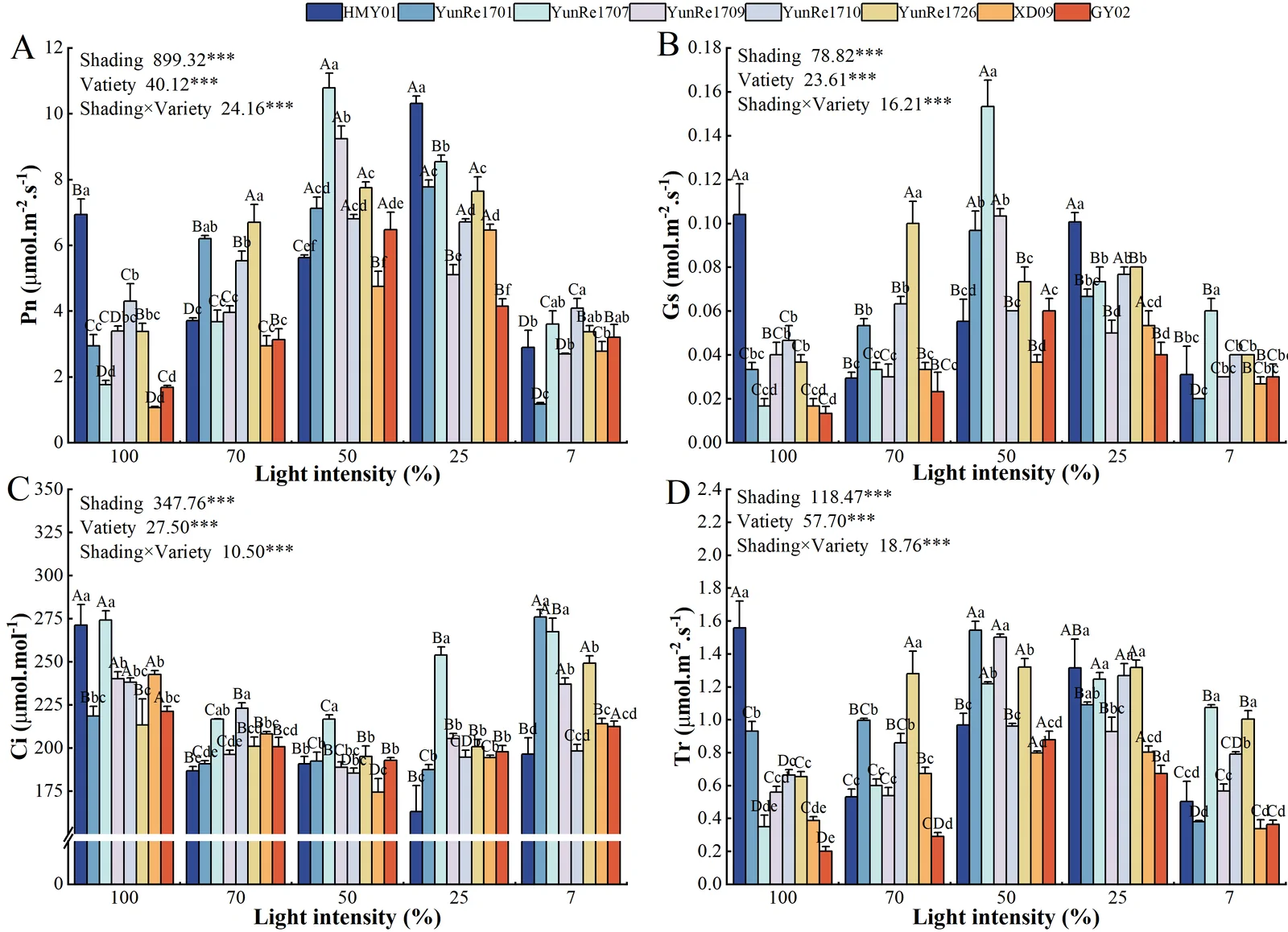
Effects of shading on leaf gas exchange parameters of different konjac varieties. **(A)** net photosynthetic rate (Pn), **(B)** stomatal conductance (Gs), **(C)** intercellular CO_2_ concentration (Ci), **(D)** transpiration rate (Tr). Different uppercase letters indicate significant differences among different light treatments for the same variety (*P* < 0.05); different lowercase letters indicate significant differences among different varieties under the same light treatment (*P* < 0.05). *** indicates significance at the *P* < 0.001 level, and the numbers represent the *F*-values. Data are presented as means ± SE (n = 3).

Two-way ANOVA revealed that shading, variety, and their interaction all had highly significant effects (*P* < 0.001) on the photosynthetic gas exchange parameters of konjac leaves (**Figure 3**). With decreasing light intensity, the Pn of konjac leaves initially increased and then decreased (**Figure 3A**). For HMY01, Pn under 25% light intensity was significantly higher than under other treatments, with an increase of 48.82% compared with full light. For YunRe1701, YunRe1707, YunRe1709, YunRe1710, YunRe1726, XD09, and GY02, Pn increased under 70% light intensity and reached its maximum within the range of 25%–50% light intensity, which were significantly higher than under other treatments, with values 2.66, 6.12, 2.73, 1.58, 2.29, 6.08, and 3.86 times those under full light, respectively. The Pn of all varieties decreased under 7% light intensity, with reductions of 72.92%, 84.86%, 57.73%, 47.32%, 39.12%, 55.89%, 56.97%, and 22.85% compared with those under 25% light, respectively.

The effect of shading on Gs was consistent with that on Pn, generally showing an initial increase followed by a decrease with decreasing light intensity (**Figure 3B**). For HMY01, Gs under 100% and 25% light intensities was significantly higher than under other treatments. For YunRe1701, YunRe1707, YunRe1709, and GY02, Gs reached its maximum under 50% light intensity and then gradually decreased, with values 2.90, 9.18, 2.58, and 4.51 times those under full light, respectively. For YunRe1710 and XD09, Gs significantly increased under 70% light, reached its maximum under 25% light intensity, and then began to decrease. For YunRe1726, Gs reached its maximum under 70% light, significantly higher than under other treatments.

The Ci of konjac leaves first decreased and then increased with increasing shading intensity (**Figure 3C**). For HMY01, Ci was highest under full light and reached its minimum under 25% light intensity. For YunRe1701, Ci reached its minimum under 25% light intensity and its maximum under 7% light, with significant difference between the two. For YunRe1707, YunRe1709, YunRe1710, XD09, and GY02, Ci was highest under full light and lowest under 50% light, with reductions of 20.94%, 21.43%, 22.07%, 28.17%, and 12.85%, respectively. For YunRe1726, Ci was lowest under 50% light and highest under 7% light, with an increase of 27.70%.

The effect of shading on the Tr of konjac leaves exhibited a similar trend to that of Gs, generally showing an initial increase followed by a decrease with decreasing light intensity (**Figure 3D**). For HMY01, Tr was highest under 100% and 25% light intensities and lowest under 7% light. For YunRe1701, Tr was highest under 50% light and lowest under 7% light, with a reduction of 75.38%. For YunRe1707 and YunRe1710, Tr was lowest under 100% light and highest under 25% light. For YunRe1709, Tr was lowest under 100%, 70%, and 7% light, with no significant differences among these three; Tr was highest under 50% light, significantly higher than under other treatments. For YunRe1726 and GY02, Tr was highest under 50% light, with values 2.02 and 4.38 times those under 100% light, respectively. For XD09, Tr was highest under 25% light intensity and lowest under 7% light intensity.

#### 3.3.2 Chlorophyll fluorescence parameters

**Figure 4:**
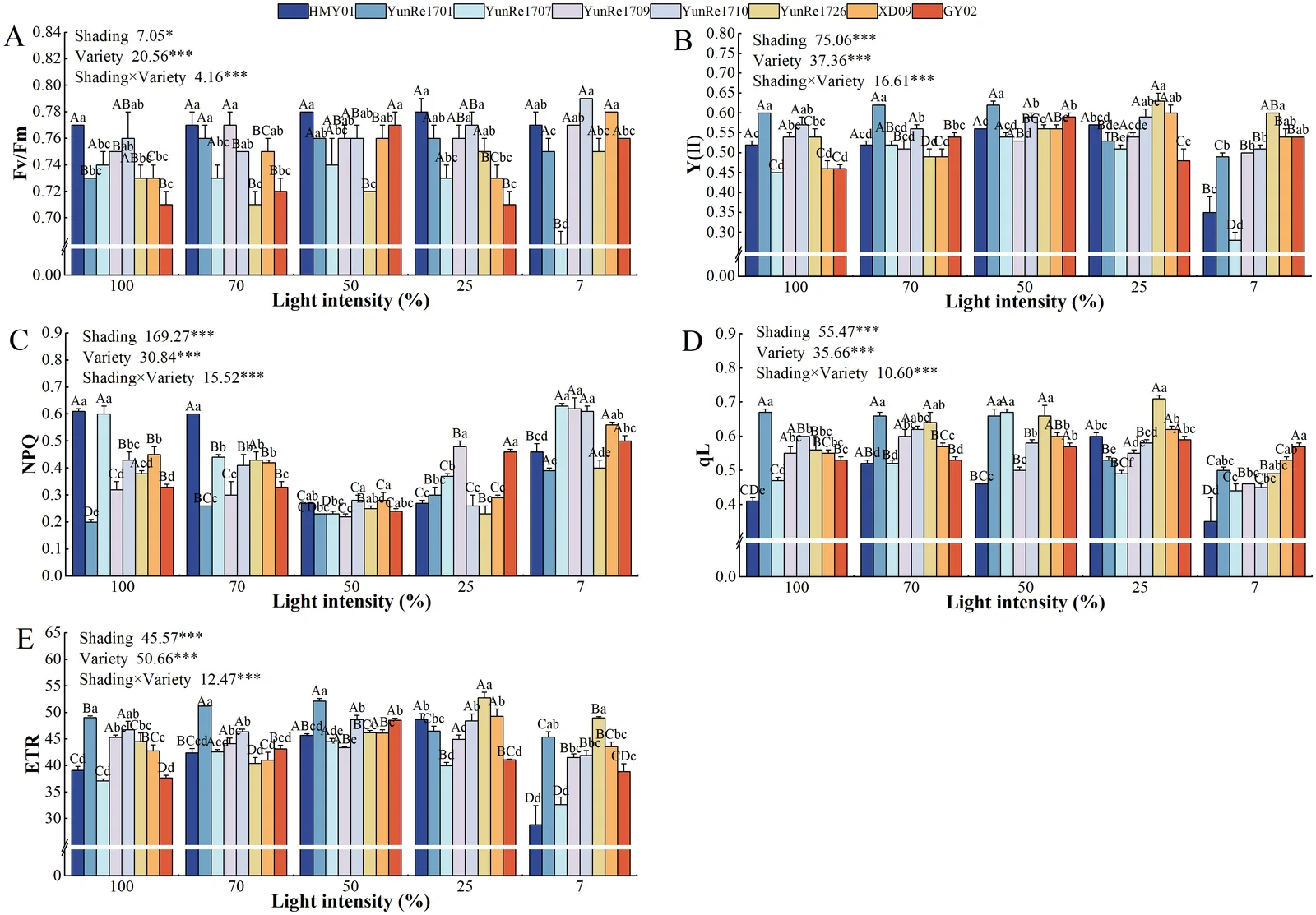
Effects of shading on chlorophyll fluorescence parameters of different konjac varieties. **(A)** maximum photochemical efficiency of photosystem II (Fv/Fm), **(B)** actual photochemical efficiency (Y(II)), **(C)** non-photochemical quenching (NPQ), **(D)** photochemical quenching (qL), **(E)** relative electron transport rate (ETR). Different uppercase letters indicate significant differences among different light treatments for the same variety (*P* < 0.05); different lowercase letters indicate significant differences among different varieties under the same light treatment (*P* < 0.05). *** indicates significance at the *P* < 0.001 level, * indicates significance at the *P* < 0.05 level. and the numbers represent the *F*-values. Data are presented as means ± SE (n = 3).

Two-way ANOVA revealed that shading, variety, and their interaction all had significant effects (*P* < 0.05) on the 5 chlorophyll fluorescence parameters of konjac leaves (**Figure 4**). The effects of shading on Fv/Fm of konjac leaves are shown in **Figure 4A**. No significant differences were observed among treatments for HMY01. For YunRe1701, Fv/Fm was lowest under 7% light intensity, significantly lower than under other treatments. For YunRe1707, YunRe1726, XD09, and GY02, Fv/Fm was lowest under 100% light. For YunRe1709 and YunRe1710, Fv/Fm was lowest under 70% light intensity. For Y(II), HMY01, YunRe1701, YunRe1707, YunRe1709, and YunRe1726 all exhibited significantly lower values under 7% light than under other treatments (**Figure 4B**). For YunRe1710, Y(II) was significantly lower under 70% light than under other treatments. For XD09 and GY02, Y(II) was lowest under 100% light. For the eight konjac varieties, NPQ first decreased and then increased with decreasing light intensity (**Figure 4C**). For HMY01, YunRe1701, YunRe1709, YunRe1710, and XD09, NPQ under 50% and 25% light was significantly lower than under other treatments. For YunRe1707 and GY02, NPQ under 50% light was significantly lower than under other treatments. With decreasing light intensity, both qL and ETR first increased and then decreased (**Figures 4D, E**). For HMY01, YunRe1701, YunRe1710, XD09, and GY02, both qL and ETR reached their maxima under 25% or 50% light.

### 3.4 Effects of shading on konjac yield

**Figure 5:**
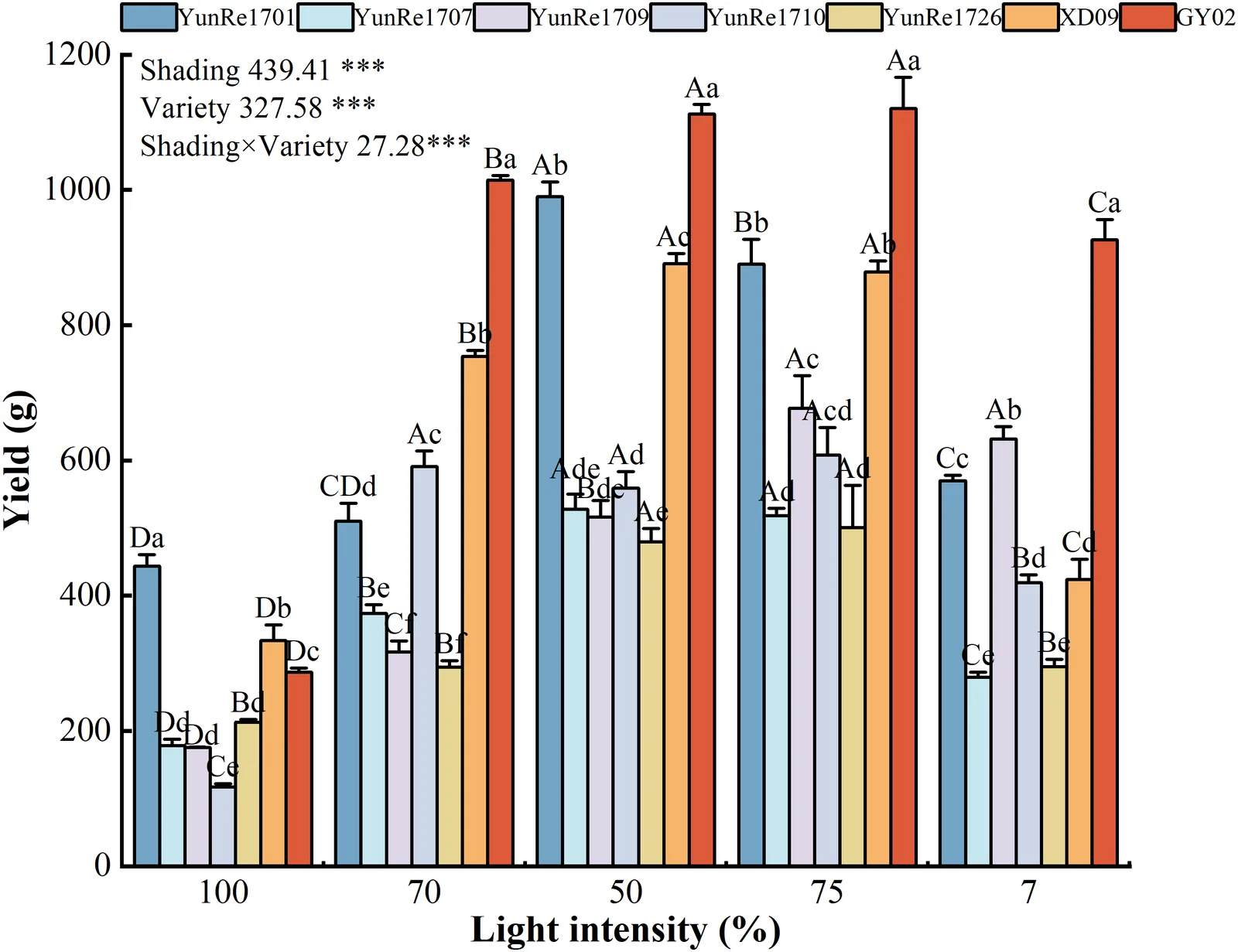
Effects of shading on corm yield of different konjac varieties. Different uppercase letters indicate significant differences among different light treatments for the same variety (*P* < 0.05); different lowercase letters indicate significant differences among different varieties under the same light treatment (*P* < 0.05). *** indicates significance at the *P* < 0.001 level, and the numbers represent the *F*-values. Data are presented as means ± SE (n = 3).

HMY01 was not included in the yield analysis because severe soft rot disease occurred during the later growth stage, resulting in complete plant mortality and no corm harvest. Two-way ANOVA revealed that shading, variety, and their interaction all had highly significant effects (*P* < 0.001) on the yield (**Figure 5**). The effect of shading on konjac yield exhibited a pattern of initial increase followed by decrease with decreasing light intensity. For YunRe1701, YunRe1707, YunRe1709, YunRe1710, YunRe1726, XD09, and GY02, yield was highest under 50% or 25% light intensity and lowest under 100% light, with increases of 123.27%, 196.05%, 286.16%, 417.40%, 135.40%, 166.86%, and 250.92%, respectively (**Figure 5**). Except for YunRe1709, the yields of the other varieties under 7% light were also significantly lower than those under 50% and 25% light (*P* < 0.05).

### 3.5 Correlations among growth traits, photosynthesis, and yield of konjac

**Figure 6:**
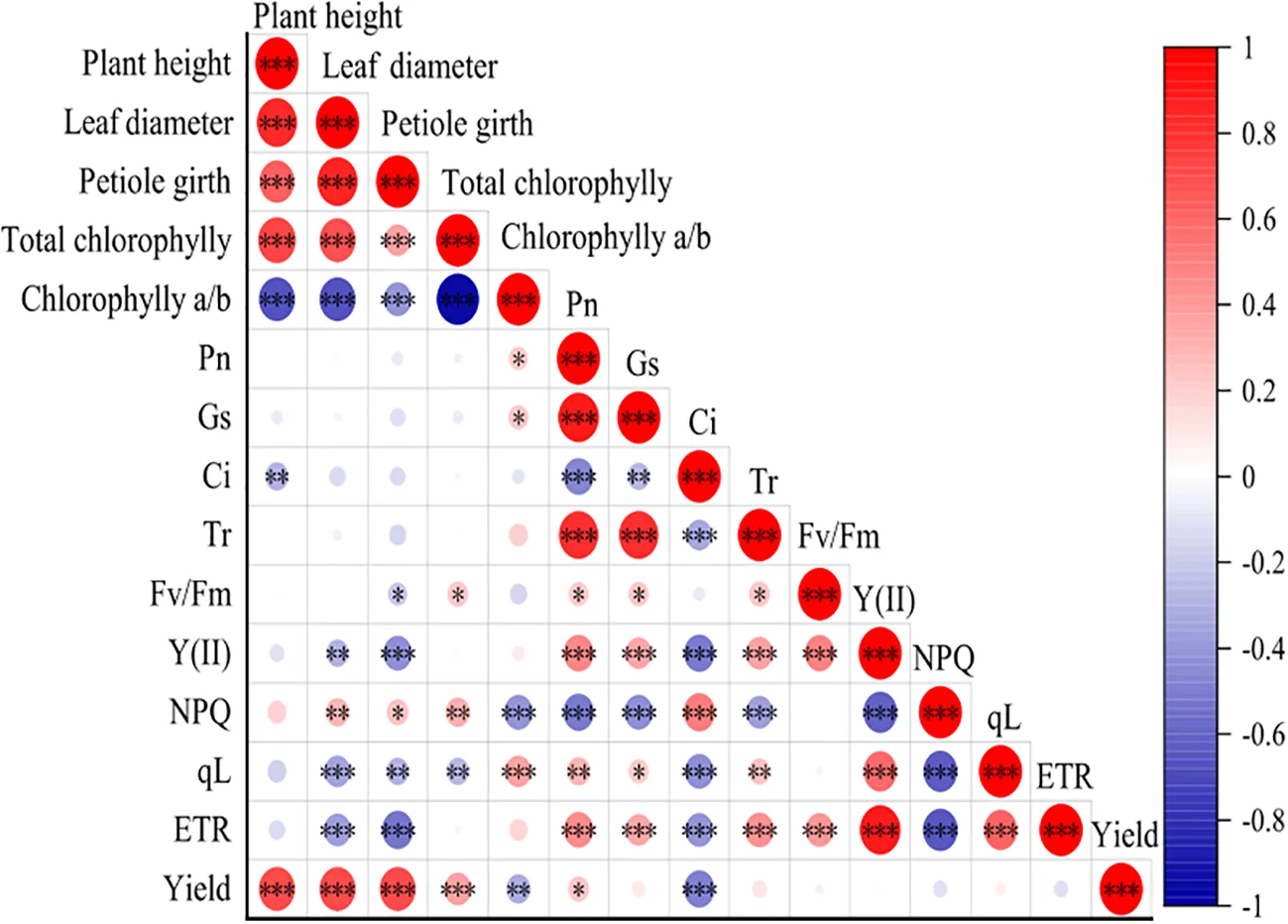
Correlation matrix of 15 measured traits. * indicates *P* < 0.05; ** indicates *P* < 0.01; *** indicates *P* < 0.001.

Correlation analysis of the 15 traits across different konjac varieties revealed that yield was strongly and positively correlated with plant height, leaf diameter, and petiole girth, with correlation coefficients reaching 0.704 or above (**Figure 6**; **Supplementary Table 2**). The correlation coefficients among plant height, leaf diameter, and petiole girth ranged from 0.64 to 0.85. Total chlorophyll content was strongly and positively correlated with plant height and leaf diameter (r = 0.74 and 0.68, respectively), and exhibited a strong negative correlation with chlorophyll a/b ratio (r = -0.95). Among the photosynthetic parameters, Pn was strongly and positively correlated with Gs and Tr (r > 0.82). NPQ showed strong negative correlations with Y(II), qL, and ETR, with correlation coefficients ranging from 0.61 to 0.66. ETR was strongly and positively correlated with Y(II) and qL, with correlation coefficients of 0.89 and 0.60, respectively.

### 3.6 Comprehensive evaluation of the effects of shading on konjac growth and physiology

To further elucidate the effects of different light intensities on the growth and physiology of konjac, principal component analysis (PCA) was performed on 14 indices (excluding yield-related traits) across the eight konjac varieties, as well as on 15 indices (including yield-related traits) across the seven *A. bulbifer* varieties (**Table 2**, **Supplementary Table 3**). Based on the criterion of eigenvalues greater than 1, four principal components (PCs) were extracted, with a cumulative contribution rate of 81.23% (**Table 2**). PC1, which accounted for 33.85% of the variance, was mainly composed of ETR, Y(II), NPQ, Pn, Gs, and Tr. PC2, which accounted for 22.42% of the variance, was mainly composed of leaf diameter, total chlorophyll content, chlorophyll a/b ratio, and plant height. PC3, which accounted for 14.01% of the variance, was mainly composed of qL, Fv/Fm, plant height, and petiole girth. PC4, which accounted for 10.96% of the variance, was mainly composed of Gs, Fv/Fm, Tr, and Y(II).

**Table 2:** Characteristic vector, eigenvalues and contribution rates from principal component analysis.

Item	Index	PC1	PC2	PC3	PC4
Eigenvector	Plant height	-0.31	0.63	-0.52	0.04
	Leaf diameter	-0.52	0.77	-0.23	0.15
	petiole girth	-0.48	0.47	-0.47	0.39
	Total chlorophylly	-0.42	0.74	0.26	-0.35
	chlorophylly a/b	0.54	-0.64	-0.26	0.35
	Pn	0.69	0.50	0.24	0.38
	Gs	0.62	0.38	0.38	0.49
	Ci	-0.37	-0.48	0.30	0.16
	Tr	0.62	0.39	0.37	0.42
	Fv/Fm	0.22	0.34	0.57	-0.46
	Y(II)	0.80	0.23	-0.12	-0.40
	NPQ	-0.73	-0.14	0.36	0.03
	qL	0.66	-0.03	-0.59	-0.13
	ETR	0.81	0.16	-0.20	-0.37
Eigenvalue		4.74	3.14	1.96	1.53
Contribution rate / %		33.85	22.42	14.01	10.96
Accumulated contribution rate / %		33.85	56.27	70.27	81.23
Weight		0.42	0.28	0.17	0.13

**Figure 7:**
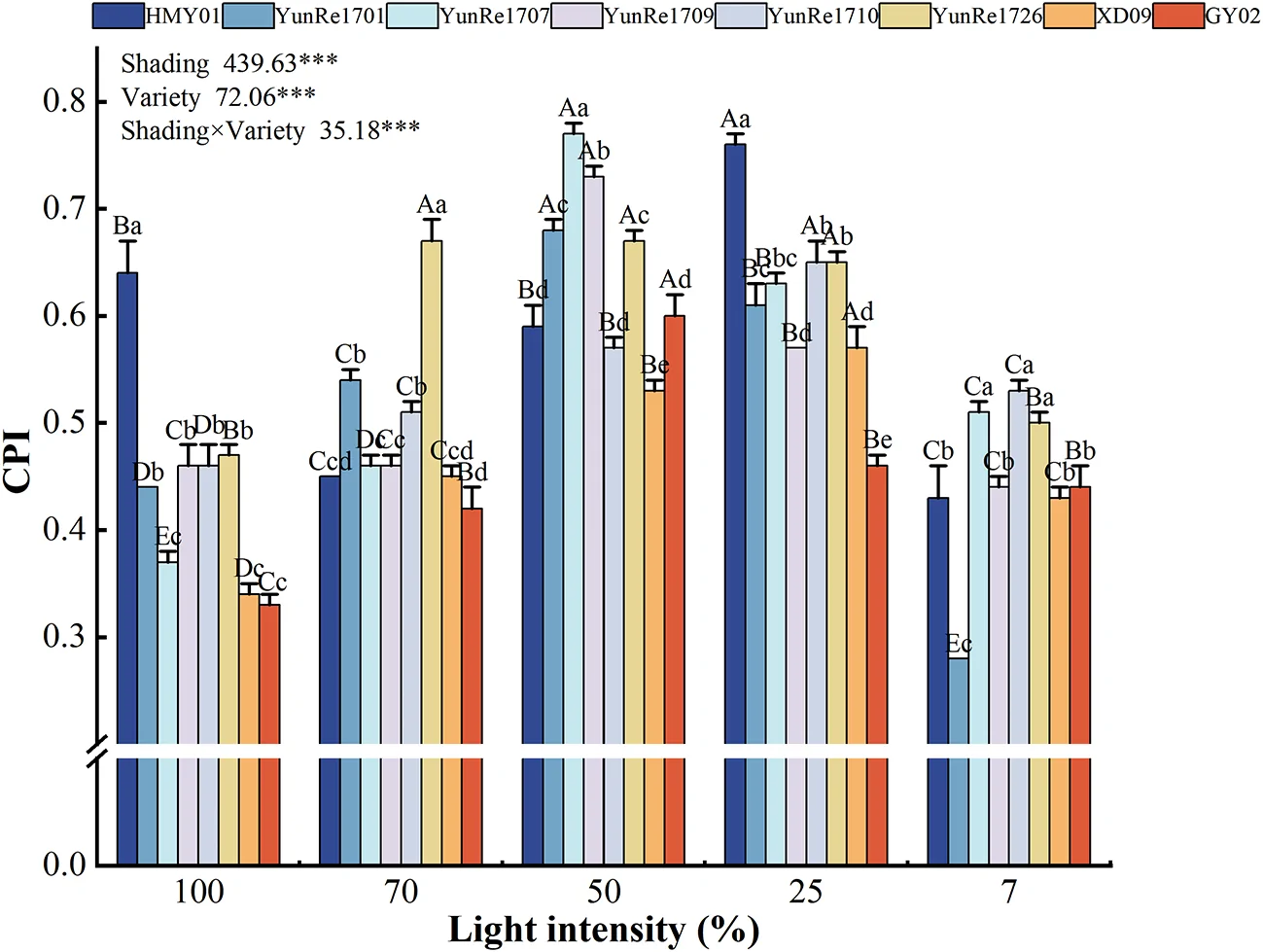
Composite index (CPI) of eight konjac varieties under different light intensities. Different uppercase letters indicate significant differences among different light treatments for the same variety (*P* < 0.05); different lowercase letters indicate significant differences among different varieties under the same light treatment (*P* < 0.05). *** indicates significance at the *P* < 0.001 level, and the numbers represent the *F*-values. Data are presented as means ± SE (n = 3).

**Figure 8:**
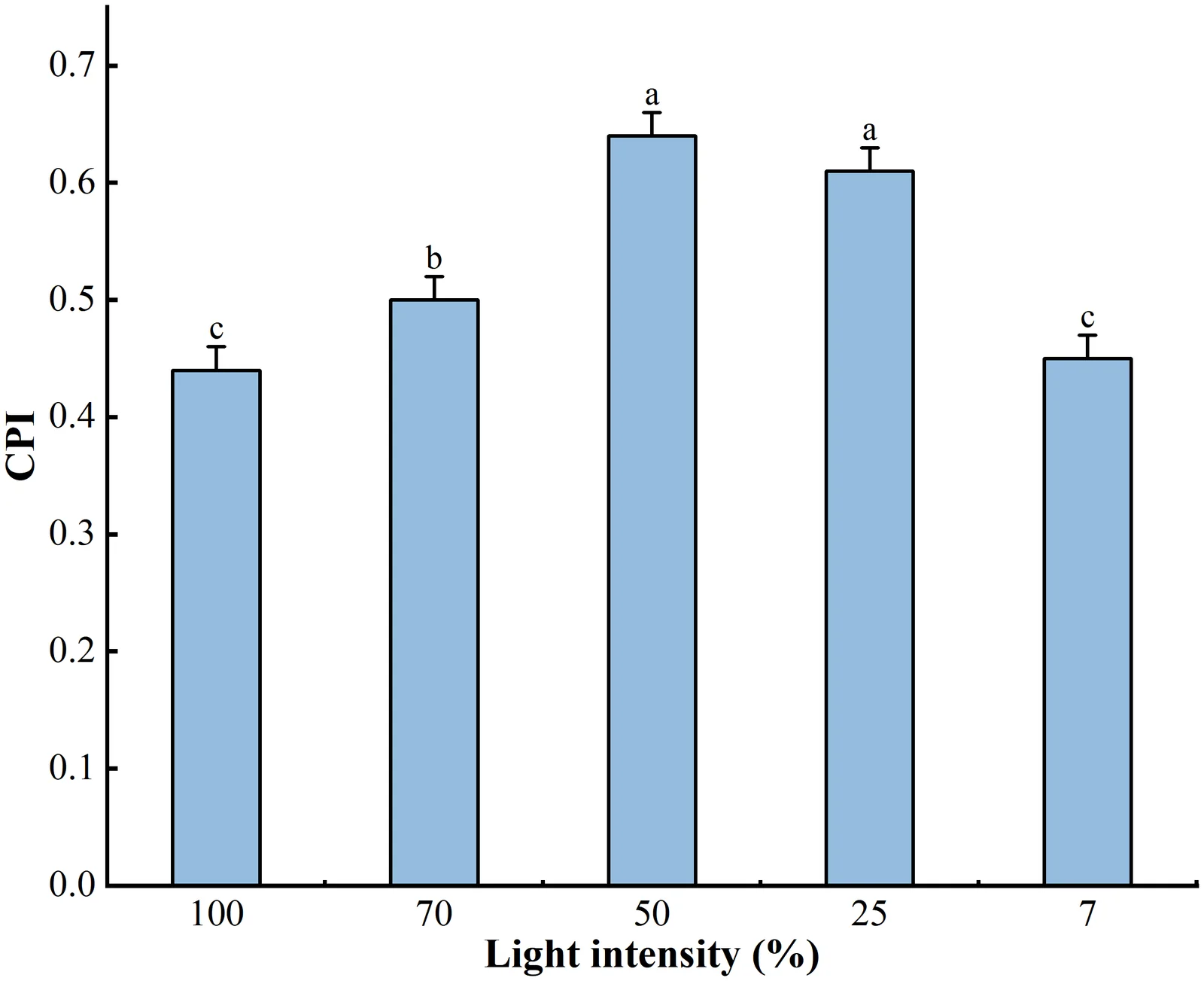
Comparison of CPI values across different light intensities. Different lowercase letters indicate significant differences among the treatment groups (*P* < 0.05). Data are presented as means ± SE (n = 3).

To systematically evaluate the effects of shading on aboveground morphology, photosynthesis, and yield of konjac, we performed a membership function analysis based on the four comprehensive index values (Y1, Y2, Y3, and Y4) of the eight varieties derived from PCA (**Supplementary Tables 4**, **5**), and calculated the composite index (CPI) for each variety under different light intensities (**Figure 7**; **Supplementary Figure 1**). For HMY01, YunRe1710, and XD09, CPI was significantly higher under 25% light than under other treatments; for YunRe1701, YunRe1707, YunRe1709, and GY02, CPI was significantly higher under 50% light than under other treatments; for YunRe1726, CPI was significantly higher under 25%–70% light than under other treatments. Comparison of the average CPI values of the eight varieties across all light treatments further confirmed that 25%–50% light intensity was more favorable for konjac growth, representing the suitable light environment for konjac (**Figure 8**; **Supplementary Figure 2**). Under 100% light intensity, HMY01 exhibited significantly higher CPI than the other varieties. Under 70% light, YunRe1726 showed significantly higher CPI than the other varieties. Under 50% light, YunRe1707, YunRe1709, YunRe1701 and YunRe1726 had relatively high CPI values. Under heavy shading (25% and 7% light), YunRe1710, YunRe1707 and YunRe1726 had the highest CPI (**Figure 7**).

**Figure 9:**
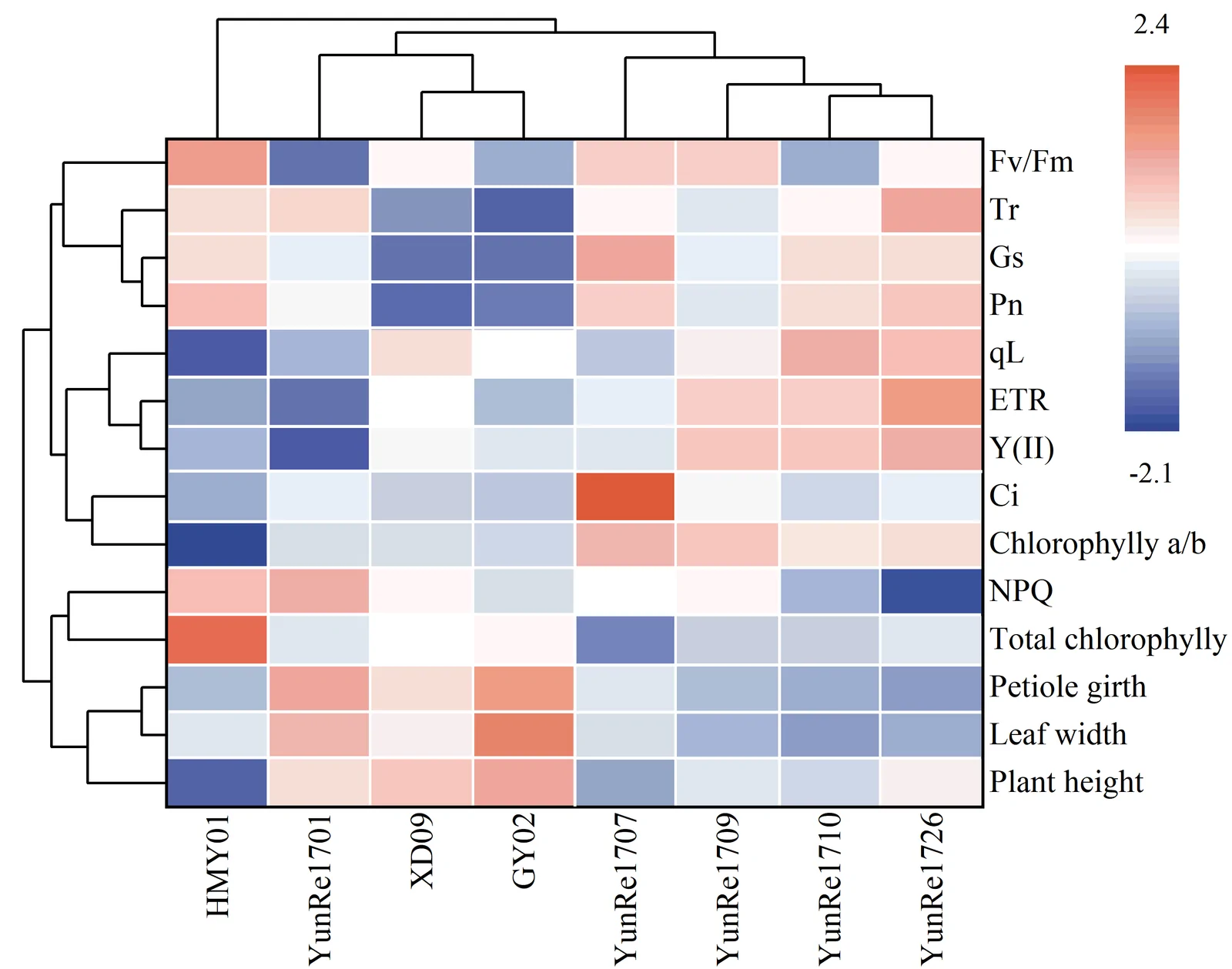
Cluster heatmap of eight konjac varieties based on morphological and photosynthetic traits.

To further characterize the morphological, photosynthetic, and yield traits of the eight konjac varieties, cluster analysis was performed. The results showed that HMY01 was distinctly separated from the other seven varieties in terms of plant height and photosynthetic traits, consistent with the botanical classification between *A. konjac* and *A. bulbifer*. YunRe1701, XD09, and GY02 were grouped into one cluster, characterized by distinct advantages in plant height, leaf diameter, and petiole girth. YunRe1707, YunRe1709, YunRe1710, and YunRe1726 were grouped into another cluster, characterized by superior photosynthetic performance (**Figure 9**; **Supplementary Figure 3**). Collectively, this study provides a scientific basis for selecting suitable light environments for konjac cultivation and for allocating konjac varieties under different light conditions.

## 4 Discussion

### 4.1 Effects of shading on morphology of different konjac varieties

Changes in light intensity first trigger morphological responses in plants, which represent the most direct and immediate strategy for adapting to alterations in the light environment. Under shading conditions, plants typically enhance light interception capacity through increased plant height and expanded leaf area (Ruberti et al., 2012; Wang et al., 2026). In this study, with increasing shading intensity, plant height, leaf diameter, and petiole girth of the eight konjac varieties generally exhibited upward trends; however, significant inter-varietal differences were observed in both the optimal light intensity and the magnitude of response. HMY01 reached its maximum plant height under 25% light, whereas most *A. bulbifer* varieties only showed significant increases under 25% light and reached their highest values under 7% light. This is consistent with the observations of Wang et al. (2024) in shade-tolerant peanut varieties, where the main stem elongation under shading was significantly smaller than that in sensitive varieties. In contrast, the magnitude of plant height increase in *A. bulbifer* varieties was generally greater than that in HMY01, which may reflect a stronger light-seeking response of *A. bulbifer* as a typical shade-demanding plant to low light. Leaf diameter showed a similar trend. For most *A. bulbifer* varieties, leaf diameter reached its highest values under 7% light, whereas that of HMY01 peaked under 25% light and then stabilized, suggesting that HMY01 reaches its morphological adjustment limit at milder shading levels. Regarding petiole girth, HMY01, YunRe1701, and XD09 exhibited a unimodal response (peaking under 50% light), whereas YunRe1709, YunRe1710, YunRe1726, and GY02 reached their maximum petiole girth under 7% light. Overall, under shading conditions, konjac primarily responded to reduced light intensity through increased plant height, leaf expansion, and petiole thickening (Niinemets et al., 2007). Lü et al. (2023) also demonstrated in their evaluation of shade tolerance in sweet potato that morphological traits such as vine length, leaf area index, and branch number are important dimensions for assessing shade tolerance. However, when light intensity fell below the adaptive range for a given variety, plant height growth stagnated, leaf area decreased, and petiole girth no longer thickened (Zhan et al., 2020), as observed in HMY01, further revealing the differentiation of morphological adaptation strategies among varieties.

### 4.2 Effects of shading on photosynthetic pigments and photosynthetic gas exchange parameters

Changes in chlorophyll content represent a classic physiological response of plants to variations in the light environment. Previous studies have shown that with increasing shading intensity, total chlorophyll content in leaves increases while the chlorophyll a/b ratio decreases, which is consistent with the findings of the present study (Li et al., 2023; Guo et al., 2010). This may reflect a mechanism by which plants enhance light capture through the accumulation of more chlorophyll, particularly chlorophyll b, thereby improving leaf adaptability to low-light environments (Gong et al., 2015; Yang et al., 2024). Li et al. (2024a) suggested that higher chlorophyll content and lower chlorophyll a/b ratio could serve as important indicators for evaluating crop shade tolerance. In the present study, significant differences in response thresholds were observed among the eight konjac varieties: total chlorophyll content of HMY01 significantly increased under 70% light, whereas most *A. bulbifer* varieties showed significant increases under 25%–50% light. This difference indicates that HMY01 (*A. konjac*) exhibits a more ‘sensitive’ adjustment of photosynthetic pigments, initiating compensatory mechanisms under mild shading, while *A. bulbifer* varieties demonstrate stronger low-light tolerance. This is consistent with the findings of Li et al. (2024b) in wheat under shading, where shade-tolerant varieties (MM-51 and CM-39) adapted to low-light conditions through higher chlorophyll content. These distinct pigment response thresholds may help explain why the varieties cluster into different functional groups.

The response of Pn to shading followed a unimodal curve, all eight konjac varieties reached their Pn peak under 25%–50% light, with a sharp decline under 7% light, indicating that extreme low light exceeded the adaptive limits of the photosynthetic apparatus of konjac. Moreover, the fact that all varieties shared the same optimal light range for Pn, yet differed in the magnitude of response, suggests that while the fundamental photosynthetic capacity is conserved across varieties, the efficiency of light utilization and the ability to sustain photosynthesis under suboptimal conditions vary considerably. The trends of Gs and Tr were highly consistent with that of Pn, while Ci showed an initial decrease followed by an increase, suggesting that the primary limiting factor of photosynthesis was non-stomatal (Du et al., 2011). This pattern is in close agreement with the findings of Li et al. (2024b) in wheat: under shading stress, stomatal conductance and carboxylation efficiency decreased, while intercellular CO_2_ concentration increased, indicating that Rubisco-mediated non-stomatal limitation is the dominant factor responsible for the reduction of net photosynthetic rate under low-light conditions.

### 4.3 Effects of shading on PSII photochemical efficiency and photoprotective mechanisms

Chlorophyll fluorescence parameters provide real-time information on the functional status of PSII. In this study, Fv/Fm was lowest under 100% or 7% light in most varieties, while Y(II), qL, and ETR first increased and then decreased with increasing shading intensity, reaching their maxima under 25% or 50% light. This indicates that moderate shading alleviates PSII photoinhibition pressure and improves photochemical efficiency, whereas both high-light stress under full light and extreme low light under 7% light impair PSII function. Zhang et al. (2023) also demonstrated in their study on *Keteleeria fortunei* var. *cyclolepis* seedlings that shading significantly affects chlorophyll fluorescence parameters, with moderate shading favoring seedling growth. Notably, significant differences in sensitivity to light stress were observed among varieties: HMY01 exhibited no significant differences in Fv/Fm across treatments, indicating relatively strong PSII stability, whereas the seven *A. bulbifer* varieties showed the lowest Fv/Fm under 100% or 70% light, suggesting greater sensitivity to high light. Zhang et al. (2024) also found significant differences in photosynthetic and fluorescence characteristics among sweet potato varieties with different low-light tolerances under low-light conditions. The distinct PSII stability between HMY01 and *A. bulbifer* varieties likely reflects different evolutionary adaptations to light environments. HMY01 may have originated from more open habitats where exposure to high light is frequent, leading to the evolution of robust PSII photoprotection mechanisms. Conversely, *A. bulbifer* varieties may have evolved in more shaded understory environments where high-light exposure is rare, rendering their PSII centers more vulnerable to photoinhibition when suddenly exposed to high light. This differential susceptibility to photoinhibition likely contributed to the functional grouping of varieties in the cluster analysis, where HMY01 separated distinctly from the *A. bulbifer* varieties.

NPQ is an important indicator of thermal dissipation capacity in plants. In this study, NPQ was lowest under 50% and 25% light and higher under 7% and 100% light. This U-shaped response pattern, which initially decreases and then increases, reveals the dual characteristics of photoprotective strategies in konjac: under moderate shading, light energy supply and photochemical demand tend to be balanced, reducing the need for thermal dissipation; under full light (high-light stress) and 7% light (extreme low light resulting in impaired photosynthetic electron transport), the accumulation of excess excitation energy forces plants to activate thermal dissipation mechanisms to protect PSII. This finding is consistent with the observations of Wang and Zhang (2026) on the photosynthetic physiological responses of three *Polygonum* species to shading, indicating that under moderate shading, plants activate their own protective regulatory mechanisms by increasing Fv/Fm, Y(II), qL, and ETR to enhance electron transport and light energy absorption while reducing NPQ, thereby supporting their own growth (Lu et al., 2023; Jia et al., 2015).

### 4.4 Effects of shading on yield and the morphology-photosynthesis-yield coupling relationship

Yield is a comprehensive reflection of the accumulation and distribution of photosynthetic products in plants. In this study, the yields of the seven varieties all exhibited peaking under 50% or 25% light, indicating that moderate shading exerted a pronounced promoting effect on konjac yield. This result is consistent with the findings of Qin et al. (2019) in *A. konjac* cv. No.1 Chumohua, where corm weight was highest (172.24 g) under 50%–70% shading and lowest (106.46 g) under full light. Peng et al. (2024) also demonstrated that photosynthetic performance and yield under ginger-citrus intercropping and ginger monoculture with shading were higher than those under ginger monoculture without shading. The substantial variation in yield increase among *A. bulbifer* varieties (from 123.27% in YunRe1701 to 417.40% in YunRe1710) suggests marked differences in their capacity to translate improved light conditions into harvestable yield. YunRe1710, which showed the highest yield increase, also exhibited superior photosynthetic performance and moderate morphological adjustment, indicating that a combination of photosynthetic efficiency and appropriate morphological plasticity may be the optimal strategy for maximizing yield under shaded conditions. In contrast, varieties that relied predominantly on morphological expansion (such as YunRe1701 and XD09) showed more moderate yield gains, suggesting that excessive morphological investment may come at the cost of reduced carbohydrate allocation to corms.

Correlation analysis further revealed the coupling relationship among morphology, photosynthesis, and yield, this result indicates that under moderate shading, morphological expansion (increasing light capture area) and photosynthetic enhancement (improving carbon assimilation efficiency) are closely associated with increased yield, suggesting a possible synergistic source-sink relationship among these traits (Yu et al., 2015). PCA condensed the 14 indices into four principal components. PC1 reflected the synergistic dimension of ‘light energy utilization and carbon assimilation’, while PC2 represented the core dimension of ‘morphological construction and pigment adjustment’. This multi-dimensional structure indicates that the shade tolerance of konjac varieties cannot be assessed by a single indicator but requires the integration of multiple dimensions, including morphology, photosynthesis, and yield (Liu et al., 2020; Wu et al., 2022). Zhang et al. (2025a), Hou et al. (2024), and Yuan et al. (2025b) combined principal component analysis, membership function analysis, and cluster analysis to conduct comprehensive evaluations of shade tolerance, drought tolerance, and salt tolerance in germplasm resources of tartary buckwheat, quinoa, and soybean, respectively, and successfully screened out several superior parental lines.

The CPI-based cluster analysis classified the eight varieties into three major groups: HMY01 alone (as *A. konjac*), YunRe1701, XD09, and GY02 into one group (morphology-dominant type, characterized by distinct advantages in plant height, leaf diameter, petiole girth, and yield), and YunRe1707, YunRe1709, YunRe1710, and YunRe1726 into another group (photosynthesis-dominant type, characterized by superior photosynthetic performance). This classification is consistent with previous studies on shade-tolerance typing in other crops. Wang et al. (2024) classified peanut varieties into shade-tolerant and shade-sensitive types. Zhang et al. (2025b) found significant differences in yield among seven peanut varieties under shading, with varieties such as Yuhua 22 and Yuhua 23 showing stronger tolerance to shading, while varieties such as Huayu 20 and Puhua 28 were more sensitive in terms of yield traits. The differentiation between morphology-dominant and photosynthesis-dominant types likely reflects the combined effects of evolutionary adaptation and artificial selection. Morphology-dominant varieties may have originated from environments where light competition favors rapid vertical growth and leaf area expansion, investing photosynthates in aboveground structures. In contrast, photosynthesis-dominant varieties may have evolved in environments with stable but limited light availability, favoring efficient light use over costly morphological investment. Artificial selection during domestication and breeding may have further amplified these differences. The wide variation in yield response among *A. bulbifer* varieties, particularly the exceptional performance of YunRe1710 under low light, suggests that significant genetic diversity exists within this species, which could be exploited for breeding programs targeting shaded environments. It should be noted that the proposed ‘morphology-dominant’ and ‘photosynthesis-dominant’ types are descriptive functional groupings based on PCA and cluster analysis of morphological and photosynthetic indices, rather than strict physiological or genetic classifications. Future studies integrating transcriptomic, metabolomic, or enzymatic analyses will help further elucidate the molecular mechanisms underlying these two strategies.

## 5 Conclusion

This study reveals the differentiation of shade-tolerance strategies among konjac varieties. *A. konjac* (HMY01) is relatively tolerant to high light and maintains moderate photosynthetic capacity and PSII stability under full light, whereas *A. bulbifer* varieties exhibit typical shade-loving characteristics, with optimal performance under 25%–50% light, while both full light and 7% extreme low light inhibit their growth. Light intensity of 25%–50% of natural light was identified as the suitable range for most varieties, providing a reference for light transmittance regulation in understory intercropping systems. The multi-indicator comprehensive evaluation system (PCA, membership function analysis, and cluster analysis) provides a methodological reference for breeding shade-tolerant varieties.

## Data Availability

The original contributions presented in the study are included in the article/**Supplementary Material**. Further inquiries can be directed to the corresponding authors.
