# Supplementary material for: Effects of shading on morphology, photosynthetic characteristics, and yield of different Amorphophallus varieties
Source: Front Plant Sci. 2026 Aug 24;17:1931411. doi: 10.3389/fpls.2026.1931411 (PMC13547013; doi:10.3389/fpls.2026.1931411)
Supplement: Supplementary file 1 [file DataSheet1.docx]

Supplementary Figure 1 Composite index (CPI) of seven *A. bulbifer* varieties under different light intensities. Different uppercase letters indicate significant differences among different light treatments for the same variety (*P* < 0.05); different lowercase letters indicate significant differences among different varieties under the same light treatment (*P* < 0.05). Data are presented as means ± SE (n = 3).

Supplementary Figure 2 Comparison of CPI values across different light intensities (for seven *A. bulbifer* varieties). Different lowercase letters indicate significant differences among the treatment groups (*P* < 0.05). Data are presented as means ± SE (n = 3).

Supplementary Figure 3 Cluster heatmap of the seven *A. bulbifer* varieties.
